# A sustainable process for the recovery of volatile constituents from *Gracilaria lemaneiformis* in agar production and evaluation of their antioxidant activities

**DOI:** 10.1186/s13065-019-0590-y

**Published:** 2019-05-31

**Authors:** Shengliang Yuan, Kefeng Wu, Zhihong Duan, Yanxia Huang, Yingnian Lu, Xiaoli Ma

**Affiliations:** 1 Guangdong Key Laboratory for Research and Development of Natural Drugs, Guangdong Medical University, Zhanjiang, 524023 China; 2 Affiliated Hospital, Guangdong Medical University, Zhanjiang, 524023 China

**Keywords:** *Gracilaria lemaneiformis*, Volatile constituents, Sustainable extraction, Antioxidant, GC–MS

## Abstract

**Background:**

*Gracilaria lemaneiformis* is a common red alga used as a raw material source for the agar industry. Its extract is rich in natural volatile constituents (VCs) having antioxidant activities. Herein, a sustainable method was used to recover VCs from the alga. The chemical composition of VCs present in the *n*-hexane fraction was analyzed by gas chromatography–mass spectroscopy (GC–MS) and the antioxidant potential was measured using a series of in vitro biochemical assays, including DPPH, hydroxyl, and superoxide radical scavenging assays.

**Results:**

The recovery yield of the VCs was 0.823 wt% of the dry mass of *G. lemaneiformis*. A total of 25 VCs were successfully identified, comprising approximately 99.94% of the total volume. The major component was *n*-hexadecanoic acid (38.57%), followed by oleic acid (25.48%), arachidonic acid (12.84%), and tetradecanoic acid (2.52%). In addition, The VCs displayed strong free radical scavenging activity in the DPPH (IC_50_ = 21.56 mg/L), hydroxyl (IC_50_ = 18.34 mg/L), and superoxide (IC_50_ = 391.12 mg/L) radical scavenging assays. The antioxidant activities of the VCs exhibited a dose-dependence at concentrations ranging from 5 to 200 mg/L.

**Conclusion:**

The results indicated that the sustainable process improved the agar quality and that the extract contained many natural VCs with antioxidant activities, which have the potential to be used in functional food and cosmetics instead of as a discarded byproduct of the agar industry.

## Introduction

*Gracilaria lemaneiformis* (*G. lemaneiformis*) a species of *Gracilariales*, is abundantly cultivated along the coast of China due to its high commercial value [1]. This seaweed is edible as a sea-flavor vegetable [2], and is also used as a traditional Chinese medicine because of its abundant ingredients beneficial for human health [3, 4]. More importantly, this seaweed is used in the agar industry as a raw material because of its high polysaccharide content [5, 6]. In current agar production, dried seaweed is used as a raw material to produce agar via several methods including alkali pretreatment or acid hydrolysis [7, 8], and the volatile constituents (VCs) of *G. lemaneiformis* are destroyed and discarded as waste. The VCs of *G. lemaneiformis* are reported to exhibit cytotoxic activity and tyrosine phosphatase 1B inhibitory activity [9, 10], suggesting that these VCs could be useful in the food, cosmetic, and pharmaceutical industries [11, 12]. Therefore, it is worthwhile to recover VCs to improve the economic value of *G. lemaneiformis*.

VCs are composed of significant amounts of lipids that exhibit biological activities such as antioxidant, antitumor, anti-Rhizopus activities [13–15]. For these applications, natural antioxidants have attracted significant research attention. Compared to synthetic antioxidants, safe and inexpensive natural antioxidants are more popular with consumers. Many studies have reported that antioxidant compounds are effective for protecting human health and food safety against the oxidizing reactions by reactive oxygen species (ROS). Excessive production of ROS causes oxidative stress which damages cell structure, leading to a number of chronic diseases including stroke, cancer, diabetes, atherosclerosis, and other degenerative diseases [16, 17]. Therefore, there is significant demand for effective natural antioxidant VCs that exhibit strong antioxidant activity to eliminate the ROS and other free radicals. However, few studies have investigated the VCs from the edible seaweed as important natural antioxidants and flavor agents in food industry.

In this study, a sustainable method was developed for the recovery of VCs from the red algae *G. lemaneiformis*. The chemical composition of VCs present in the *n*-hexane fraction was analyzed by GC–MS: gas chromatography–mass spectroscopy. In addition, the antioxidant activities of the extracted VCs were evaluated by a series of biochemical assays in vitro including the DPPH, hydroxyl, and superoxide radical scavenging assays. The sustainable extraction process of VCs improved agar quality and reduced the amount of effluent, providing a source of natural antioxidants rather than waste.

## Methods

### Biological material

Red seaweed, *G. lemaneiformis*, was harvested in June 2016 from the Zhanjiang Naozhou coastline of Guangdong province (N 21°12′; E 110°4′) in Southern China. The seaweed was washed thoroughly with tap water to remove salt and impurities and subsequently air-dried at 60 °C. The dried sample was cut and mixed with a blender and stored in a refrigerator at 4 °C until use. The sample was identified by the School of Marine Science, Guangdong Ocean University, China.

### Chemicals and reagents

1,1-Diphenyl-2-picrylhydrazyl (DPPH) was purchased from Tokyo Kasei Kogyo Co., Ltd. (Tokyo, Japan). The hydroxyl radical and superoxide anion detection kits were obtained from the Nanjing Jiancheng Bioengineering Institute (Nanjing, China). Deionized water was supplied by a Milli-Q water purification system from Millipore (USA). All other reagents used in this study were of analytical grade.

### Sustainable extraction for the recovery of VCs

The natural VCs were extracted as previously described [10] with some modifications. Briefly, the dry *G. lemaneiformis* (150 g) was mixed with 70% ethanol (3000 mL) and ultrasonically pretreated 10 min, and then the mixture was refluxing at 80 °C for 2 h. After twice extractions, the extracts were combined and concentrated using a rotary evaporator at 40 °C. The crude extract was dissolved in deionized water and fractioned with *n*-hexane. Subsequently, the *n*-hexane fraction was concentrated using a rotary evaporator to recovery the solvent and obtain the VCs.

### Analysis of VCs by GC–MS

The chemical constituents of the VCs from *G. lemaneiformis* were determined via GC–MS using a standard procedure [18]. The system was equipped with an Agilent 6890 gas chromatograph/5973 N mass selective detector (Palo Alto, Calif.) and separated using an HP-FFAP (HP-free fatty acid phase) capillary column (30 m × 0.25 mm i.d.; film thickness = 0.25 μm). Helium was used as the carrier gas at a constant 1 mL min^−1^ flow rate and the oven temperature was set at 50 °C for 5 min, raised to 230 °C at 4 °C min^−1^, and held for 20 min. The detector and injector temperatures were maintained at 250 °C. The ionizing energy of the mass selective detector was set at 70 eV, with a scanning mass range of m/z 50–500. The VCs sample (1 µL of 100 times-diluted samples in methanol) was pass through a splitless injector in manual mode. The relative percentages of the constituents of the VCs were expressed as percentages calculated from the normalized peak areas. The various chemical constituents of VCs were identified by GC retention times on a DB-5 capillary column, similarity index, and mass spectra, which were consistent with the mass spectra in the computer library (Wiley 275L program). Chromatographic peaks were checked with the mass chromatograms of the characteristic fragment ion peaks for homogeneity. The chemical structures of the dominant compounds were drawn using ACD software.

### Antioxidant activities

#### DPPH radical scavenging activity

The DPPH radical scavenging activity assay was used to determine the antioxidant activity of the isolated VCs. The assay was performed according to a method previously described with slight modifications [19]. Briefly, a sample aliquot (100 μL) was mixed with 100 μL of 0.2 mM DPPH ethanol solution and the intermixture was incubated at room temperature for 30 min. The sample absorbance was measured at 517 nm. All experiments were performed in triplicate and the percentage of scavenged DPPH was calculated using the following equation:

$${\text{DPPH scavenging activity }}\left( \% \right) \, = \, \left[ {\left( {{\text{A}}_{\text{C}} - {\text{A}}_{\text{S}} } \right)/{\text{A}}_{\text{C}} } \right] \, \times { 1}00$$

where A_c_ is the absorbance value of the control (mixture of 100 μL DPPH solution and 100 μL ethanol) and A_s_ is the absorbance value of the sample. Vitamin C was used as a positive reference.

#### Hydroxyl radical scavenging activity

Hydroxyl radical (HOR) scavenging activity of the VCs was determined according to a previously described method with some modification [19]. Briefly, 1.0 mL of a ferrous sulfate solution (6.0 mM), 1.0 mL hydrogen peroxide (6.0 mM), 0.5 mL salicylic acid (2.0 mM), and 1.0 mL sample solutions (at various concentrations) were thoroughly mixed and incubated at room temperature for 40 min. The mixture absorbance was then measured at 550 nm and vitamin C was used as a positive control. The hydroxyl radical scavenging activity was calculated using the following equation:

$${\text{HOR scavenging activity }}\% \, = \, \left[ {\left( {{\text{A}}_{ 1} - {\text{A}}_{ 2} } \right) \, / \, \left( {{\text{A}}_{ 1} - {\text{A}}_{0} } \right)} \right] \, \times { 1}00;$$

where A_0_ is the absorbance value of the reagent blank, A_1_ is the positive control absorbance, and A_2_ is the absorbance value of the sample.

#### Superoxide radical scavenging activity

The superoxide anion radical (SOAR) scavenging activity of the VCs was measured using the procedure described previously with some modifications [20]. All solutions were prepared in 0.2 M phosphate buffer (pH 7.4). The reaction mixture consisted of 0.5 mL of the reaction buffer solution (pH 7.4), 100 μL PMS (15 μM), 100 μL NADH (73 μM), 100 μL NBT (50 μM), and 200 μL sample of various concentrations completely blended and incubated at room temperature for 30 min and the absorbance was measured at 550 nm. The control was a mixture without any sample and the assay was performed in triplicate. The superoxide anion radical scavenging activity (%) was analyzed using the following equation:

$${\text{SOAR scavenging activity }}\left( \% \right) \, = \, \left[ { 1- \left( {{\text{A}}_{\text{S}} /{\text{A}}_{\text{C}} } \right)} \right] \, \times 100,$$

Vitamin C was used as a reference.

### Statistical analysis

The data were analyzed using a one-way analysis of variance (ANOVA) followed by the Duncan’s test (SPSS 12.0, SPSS Inc., USA). The data are expressed as mean ± standard deviation. All experiments were performed in triplicate and p < 0.05 was considered statistically significant.

## Results and discussion

### Recovery yield of VCs from *G. lemaneiformis*

**Fig. 1:**
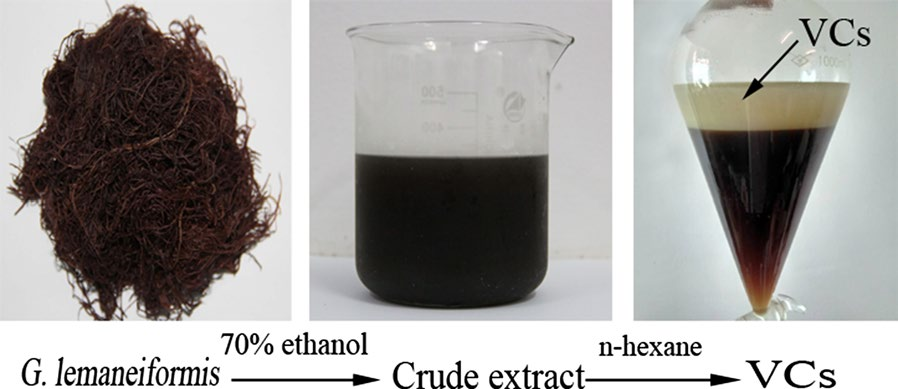
The VCs process from *G. lemaneiformis*

To manufacture high quality agar from *G. lemaneiformis*, many redundant constituents such as fatty acids, essential oils and pigments should be removed. However, it may be beneficial to recovery the VCs from the raw material seaweed as natural antioxidants. In this study, using the reflux condensing extraction shown in Fig. 1, the recovery yield of the VCs was 0.823 wt% on a dry basis from *G. lemaneiformis*. The recovery of VCs was lower for seaweeds such as *P. tenera* (1.41%) obtained by the hydro-distillation [21], but higher than that of *U. pinnatifida* (0.260%) obtained by microwave-assisted hydro-distillation [22]. The differing results of the various seaweeds can be attributed to species-specific factors, environmental differences, as well as varied extraction methods and conditions such as solvents, times, and temperatures [23] used. Therefore, it was valuable to recover and further analyze the chemical constituents of the VCs obtained from red seaweed. In this extraction process, the VCs of *G. lemaneiformis* were obtained and ethanol was recovered, the seaweed was next used as raw material to produce agar. All solvents can be recovered and recycled, this result showed no harmful pollutant was release into the environment and it was a green sustainable process.

### GC–MS analysis of VCs in the *n*-hexane fraction

**Fig. 2:**
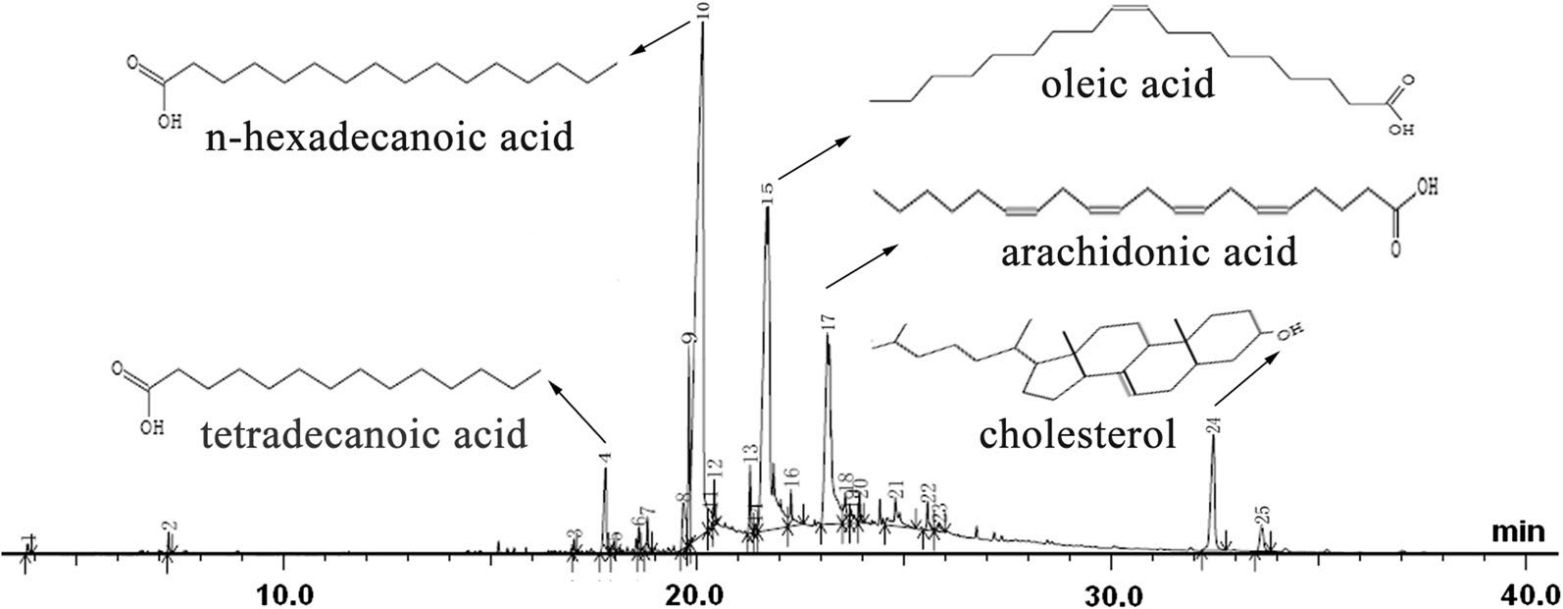
GC–MS spectra of VCs present in the *n*-hexane fraction and chemical structures of the five dominant compounds

The chemical constituents of VCs were identified via GC–MS and the results are shown in Table 1 and Fig. 2. A total of 25 volatile constituents were successfully identified comprising 99.94% of the total volume. The major constituents of the VCs obtained from *G. lemaneiformis* were *n*-hexadecanoic acid (38.57%), oleic acid (25.48%), arachidonic acid (12.84%), cholesterol (4.90%), tetradecanoic acid (2.52%). It was clear that *n*-hexadecanoic acid and tetradecanoic acid was non-reducing saturated fatty acid, in contrast, oleic acid, arachidonic acid and cholesterol was reductive.

**Table 1:** VCs identified by GC–MS in the *n*-hexane fraction of *G. lemaneiformis*

No.	RT	RPA (%)	Compound
1	3.833	0.21	Hexanal
2	7.232	0.21	Undecane
3	17.026	0.20	Heptadecane
4	17.789	2.52	Tetradecanoic acid
5	17.989	0.21	Cyclopropanecarboxylic acid, 2,2-dimethyl-3-
6	18.589	0.39	Nonadecane
7	18.801	0.53	Pentadecanoic acid
8	19.665	1.69	*cis*-9-Hexadecenoic acid
9	19.802	3.05	Dibutyl phthalate
10	20.131	38.57	*n*-Hexadecanoic acid
11	20.300	0.87	Hexadecanoic acid
12	20.408	0.57	Isopropyl palmitate
13	21.275	1.05	Phytol
14	21.392	0.25	13,16-Octadecadiynoic acid, methyl ester
15	21.716	25.48	Oleic acid
16	22.267	1.01	Isopropyl stearate
17	23.150	12.84	Arachidonic acid
18	23.583	0.99	9-Octadecenamide, (Z)-
19	23.717	0.50	Hexanedioic acid, *bis*(2-ethylhexyl) ester
20	23.933	0.46	3-Butenoic acid, 2-oxo-4-phenyl-
21	24.789	1.52	Hexadecanoic acid, 2-hydroxy-1-(hydroxymethyl
22	25.565	0.59	2-Nitrophenylcinnamamide
23	25.833	0.27	Arachidonic acid
24	32.467	4.90	Cholesterol
25	33.633	1.06	Cholest-5-en-3-ol, (3.beta.,5.alpha.)
Total identified		99.94	

The compounds were numbered in order of elution; RT, retention time (min); RPA, relative peak area

The *n*-hexadecanoic acid content was the highest (38.57%), nearly twice as high as the maximum content of *n*-hexadecanoic acid (20.08%) in *n*-hexane extracted previously from the same type of seaweed [10]. It has been reported that *n*-hexadecanoic acid exhibits various biological activities including anti-inflammatory activities, anticancer effects, and antioxidant properties [24, 25].

Tetradecanoic acid is commonly called myristic acid and is found in seaweeds such as edible kelps [26]. The tetradecanoic acid content was significant different in different alga including *G. lemaneiformis* (2.52%) (shown in Table 1), *L. japonica* (51.75%) [18], and *U. pinnatifida* (31.32%) [22]. Tetradecanoic acid has been reported to possess potential antibacterial activity [27]; it is also applied as a flavoring agent in food industry, and a brain drug additive in medicine [18].

The total amounts (43.22%) of oleic acid, arachidonic acids and cholestrol suggested that significant amounts of unsaturated ethylenic bonds (C=C) were present, therefore the VCs were imparted reductive properties. The antioxidant activity of the VCs can be ascribed to the donation of electrons to reactive oxygen radical, reducing them to more stable and non-reactive species. These polyunsaturated fatty acids represent a valuable natural source of antioxidants from seaweed extract [28, 29].

### Antioxidant activities

To determine the antioxidant effects of the VCs, a number of in vitro antioxidant assays were performed including the DPPH, hydroxyl, and superoxide radical scavenging assays. The IC_50_ values of these assays are provided in Table 2.

**Table 2:** Recovery yield and antioxidant activities of the VCs

Sample	Yield (%)	IC_50_ (mg/L)^a^		
		DPPH radical scavenging activity	Hydroxyl radical scavenging activity	Superoxide radical scavenging activity
VCs	0.823	21.56 ± 1.02	18.34 ± 1.81	391.12 ± 3.32
Vitamin C	N	4.25 ± 1.0	158.02 ± 2.65	108.28 ± 2.12

All values are mean ± SD; *SD* standard deviation

*N* not tested

^a^IC_50_: Concentration of extract (mg/L) exhibiting 50% scavenging potential

### DPPH radical scavenging activity

**Fig. 3:**
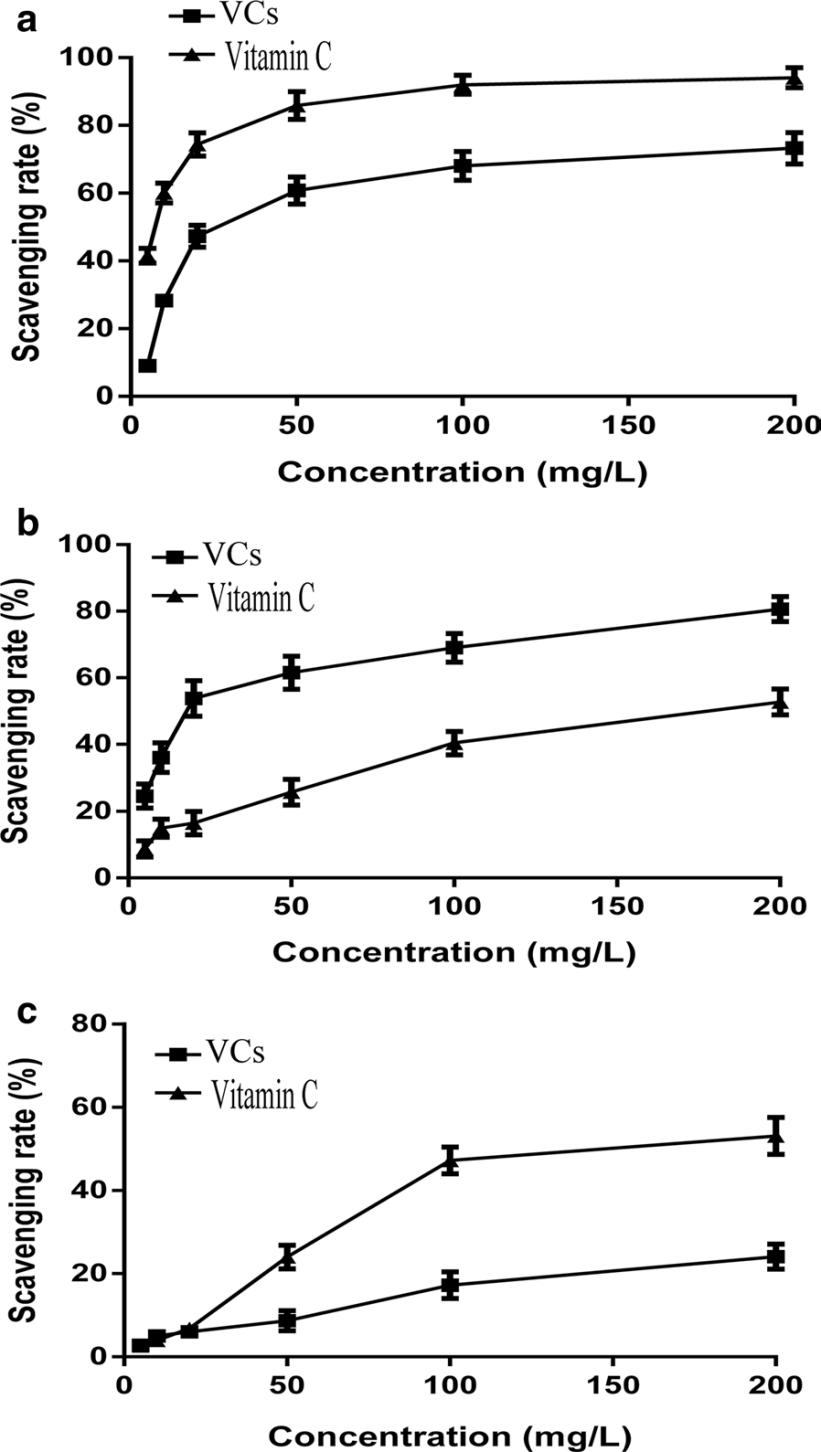
Scavenging activity of the VCs extracted from *G. lemaneiformis*. Vitamin C was used as a positive control. **a** DPPH radical, **b** hydroxyl radical, and **c** superoxide radical assays (value = mean ± SD; n = 3)

As a free radical, DPPH has been extensively used to evaluate antioxidant activity of various compounds and their free radical scavenging activity. As shown in Fig. 3a, the DPPH radical scavenging of the VCs showed dose-dependent characteristic at concentrations ranging from 5 to 200 mg/L. The VCs exhibited scavenging activity by inhibiting the DPPH radical with an IC_50_ value of 21.56 ± 1.02 mg/L, compared with that of vitamin C was 4.25 ± 1.0 mg/L. The high DPPH radical scavenging activity of VCs can be attributed to the rich unsaturated fatty acid content, which included oleic acid and arachidonic acid, which are effective DPPH free radical scavengers [30]. These result suggested that many VCs with strong antioxidant activity were present in the *G. lemaneiformis* extracts.

### Hydroxyl radical scavenging activity

The hydroxyl radical is strongly oxidizing, which can cause oxidative injury, cell damage, and death. Therefore, hydroxyl radical analysis has been widely accepted for evaluating free radical scavenging activity [31]. The hydroxyl radical scavenging potential of the VCs is shown in Fig. 3b. The VCs exhibited a concentration-dependent hydroxyl radical scavenging activity at concentrations ranging from 5 to 200 mg/L. The hydroxyl radical scavenging activity of the VCs (IC_50_ 18.34 ± 1.81 mg/L) was higher than that of the positive reference vitamin C (IC_50_ 158.02 ± 2.65 mg/L). The antioxidant activity of the VCs can be ascribed to the donation of electrons from the VCs to reactive groups, reducing them to more stable and non-reactive species. Because of their high hydroxyl radical scavenging potential, the VCs may be used in an indicator role, acting efficiently against oxidative damage caused to various biomolecules [32], and applied as a food additive or preservative.

### Superoxide radical scavenging activity

The superoxide anion is a weak oxidant and a precursor of ROS including singlet oxygen, the hydroxyl radical, and hydrogen peroxide. It can combine with other reactive species, such as nitric oxide produced by macrophages, to afford more reactive species [33]. In this study, the VCs exhibited a concentration-dependent superoxide radical scavenging activity. In addition, the VCs contained a moderate superoxide radical scavenging activity with an IC_50_ value of 391.12 ± 3.32 mg/L, which was weaker than that of obtaining from *U. pinnatifida* (IC_50_ 260.89 mg/L) [22] and the positive reference vitamin C (IC_50_ 108.28 ± 2.12 mg/L). As shown in Fig. 3c, the superoxide radical scavenging potential of the VCs from *G. lemaneiformis* were indicative of their effective antioxidant activity.

## Conclusion

In conclusion, VCs were recovered from the edible seaweed *G. lemaneiformis* commonly used in agar production using sustainable methods. In addition, the chemical constituents of the VCs were identified via GC–MS and shown to contain high contents of fatty acids, unsaturated fatty acids, aldehydes, sterols, and other types of beneficial compounds. The VCs exhibited antioxidant potential in terms of DPPH, hydroxyl, and superoxide radical scavenging activities. Thus, the VCs are effective natural antioxidants that can be utilized in various applications rather than discarded as agar industrial waste.

## Abbreviations

*G. lemaneiformis*: *Gracilaria lemaneiformis*
VCs: volatile constituents
GC–MS: gas chromatography–mass spectroscopy
ROS: reactive oxygen species
DPPH: 1,1-diphenyl-2-picrylhydrazyl
HOR: hydroxyl radical
SOAR: superoxide anion radical
RT: retention time
RPA: relative peak area

## Availability of data and materials

The datasets used and/or analysed during the current study are available from the corresponding author on reasonable request.

## References

[1] Jin M, Liu H, Hou Y, Chan Z, Di W, Li L, Zeng R (2017). Preparation, characterization and alcoholic liver injury protective effects of algal oligosaccharides from *Gracilaria lemaneiformis*. Food Res Int.

[2] Wen X, Peng C, Zhou H, Lin Z, Lin G, Chen S, Li P (2006). Nutritional composition and assessment of *Gracilaria lemaneiformis Bory*. J Integr Plant Biol.

[3] Fan Y, Wang W, Song W, Chen H, Teng A, Liu A (2012). Partial characterization and anti-tumor activity of an acidic polysaccharide from *Gracilaria Lemaneiformis*. Carbohydr Polym.

[4] Fan YL, Wang WH, Chen HS, Liu N, Liu AJ (2012). In vitro and in vivo immunomodulatory activities of an acidic polysaccharide from *Gracilaria lemaneiformis*. Adv Mat Res.

[5] Meena R, Chaudhary JP, Agarwal PK, Maiti P, Chatterjee S, Raval HD, Agarwal P, Siddhanta AK, Prasad K, Ghosh PK (2014). Surfactant-induced coagulation of agarose from aqueous extract of *Gracilaria dura* seaweed as an energy-efficient alternative to the conventional freeze–thaw process. RSC Adv.

[6] Yuan S, Duan Z, Lu Y, Ma X, Wang S (2018). Optimization of decolorization process in agar production from *Gracilaria lemaneiformis* and evaluation of antioxidant activities of the extract rich in natural pigments. 3 Biotech.

[7] Li H, Yu X, Jin Y, Zhang W, Liu Y (2008). Development of an eco-friendly agar extraction technique from the red seaweed *Gracilaria lemaneiformis*. Bioresour Technol.

[8] Wang L, Shen Z, Mu H, Lin Y, Zhang J, Jiang X (2017). Impact of alkali pretreatment on yield, physico-chemical and gelling properties of high quality agar from *Gracilaria tenuistipitata*. Food Hydrocoll.

[9] Shaoling Y, Gang Y, Bo Q, Xianqing Y (2016). Analysis of volatile compounds of dried *Gracilaria lemaneiformis* by HS-SPME method (in China). South China Fish Sci.

[10] Guo X, Gu D, Wang M, Huang Y, Li H, Dong Y, Tian J, Wang Y, Yang Y (2017). Characterization of active compounds from *Gracilaria lemaneiformis* inhibiting the protein tyrosine phosphatase 1B activity. Food Funct.

[11] Cheng C, You-Chi H, Jun H, Shan L, Yang L, Ming-Yue Z (2011). Analysis of supercritical CO_2_ extract from *Gracilaria lemaneiformis* and its application in cigarett (in China). Tob Sci Technol.

[12] Chen K, Rios JJ, Perez-Galvez A, Roca M (2017). Comprehensive chlorophyll composition in the main edible seaweeds. Food Chem.

[13] López V, Akerreta S, Casanova E, Garcia-Mina JM, Cavero RY, Calvo MI (2007). In vitro antioxidant and anti-rhizopus activities of Lamiaceae herbal extracts. Plant Foods Hum Nutr.

[14] Wang X, Xie K, Zhuang H, Ye R, Fang Z, Feng T (2015). Volatile flavor compounds, total polyphenolic contents and antioxidant activities of a China gingko wine. Food Chem.

[15] Gallego MG, Gordon MH, Segovia FJ, Skowyra M, Almajano MP (2013). Antioxidant properties of three aromatic herbs (rosemary, thyme and lavender) in oil-in-water emulsions. J Am Oil Chem Soc.

[16] Lo´pez-Tinoco C, Roca M, Garcia-Valero A, Murri M, Tinahones FJ, Segundo C, Bartha JL, Aguilar-Diosdado M (2013). Oxidative stress and antioxidant status in patients with late-onset gestational diabetes mellitus. Acta Diabetol.

[17] Al-Gubory KH (2014). Environmental pollutants and lifestyle factors induce oxidative stress and poor prenatal development. Reprod Biomed Online.

[18] Patra JK, Das G, Baek KH (2015). Chemical composition and antioxidant and antibacterial activities of an essential oil extracted from an edible seaweed, *Laminaria japonica* L. Molecules.

[19] Cho M, Kang Il-Jun, Won M-H, Lee H-S, You S (2010). Antioxidant activities of ethanol extracts and their solvent partitioned fractions from various green seaweeds. J Med Food.

[20] Duan X, Wu G, Jiang Y (2007). Evaluation of the antioxidant properties of litchi fruit phenolics in relation to pericarp browning prevention. Molecules.

[21] Patra JK, Lee S-W, Kwon Y-S, Park JG, Baek K-H (2017). Chemical characterization and antioxidant potential of volatile oil from an edible seaweed *Porphyra tenera* (Kjellman, 1897). Chem Cent J.

[22] Patra JK, Lee S-W, Park JG, Baek K-H (2017). Antioxidant and antibacterial properties of essential oil extracted from an edible seaweed *Undaria pinnatifida*. J Food Biochem.

[23] Cho M, Lee H-S, Kang I-J, Won M-H, You S (2011). Antioxidant properties of extract and fractions from *Enteromorpha prolifera*, a type of green seaweed. Food Chem.

[24] Aparna V, Dileep KV, Mandal PK, Karthe P, Sadasivan C, Haridas M (2012). Anti-inflammatory property of *n*-hexadecanoic acid: structural evidence and kinetic assessment. Chem Biol Drug Des.

[25] Arora N, Pandey-Rai S (2014). GC–MS analysis of the essential oil of *Celastrus paniculatus* Willd. seeds and antioxidant, anti-inflammatory study of its various solvent extracts. Ind Crops Prod.

[26] Kajiwara T, Hatanaka A, Kawai T, Ishihara M, Tsuneya T (1988). Study of flavor compounds of essential oil extracts from edible Japanese kelps. J Food Sci.

[27] Seanego CT, Ndip RN (2012). Identification and antibacterial evaluation of bioactive compounds from *Garcinia kola* (Heckel) seeds. Molecules.

[28] Horincar VB, Parfene G, Tyagi AK, Gottardi D, Dinică R, Guerzoni ME, Bahrim G (2013). Extraction and characterization of volatile compounds and fatty acids from red and green macroalgae from the Romanian Black Sea in order to obtain valuable bioadditives and biopreservatives. J Appl Phycol.

[29] Mohy El-Din SM, El-Ahwany AMD (2016). Bioactivity and phytochemical constituents of marine red seaweeds (*Jania rubens*, *Corallina mediterranea* and *Pterocladia capillacea*). J Taibah Univ Sci.

[30] Patra JK, Kim SH, Baek K-H (2015). Antioxidant and free radical-scavenging potential of essential oil from *Enteromorpha linza* L. prepared by microwave-assisted hydrodistillation. J Food Biochem.

[31] Qiao D, Ke C, Hu B, Luo J, Ye H, Sun Y, Yan X, Zeng X (2009). Antioxidant activities of polysaccharides from *Hyriopsis cumingii*. Carbohydr Polym.

[32] Sowndhararajan K, Kang SC (2013). Free radical scavenging activity from different extracts of leaves of Bauhinia vahlii Wight & Arn. Saudi J Biol Sci.

[33] Souza BWS, Cerqueira MA, Bourbon AI, Pinheiro AC, Martins JT, Teixeira JA, Coimbra MA, Vicente AA (2012). Chemical characterization and antioxidant activity of sulfated polysaccharide from the red seaweed *Gracilaria birdiae*. Food Hydrocoll.

